# Radiotherapy for Canine Apocrine Gland Anal Sac Adenocarcinoma: Survival Outcomes and Side Effects of a Palliative Treatment Protocol of 20 Gy in Five Consecutive Fractions

**DOI:** 10.3390/vetsci11050219

**Published:** 2024-05-15

**Authors:** Carlos Roberto Mendez Valenzuela, Kelly Shin, Hsin-Yi Weng, Jeannie M. Plantenga, Isabelle F. Vanhaezebrouck

**Affiliations:** 1 Department of Veterinary Clinical Sciences, College of Veterinary Medicine, Purdue University, West Lafayette, IN 47907, USA; 2 College of Science, Purdue University, West Lafayette, IN 47907, USA; 3 Department of Comparative Pathobiology, College of Veterinary Medicine, Purdue University, West Lafayette, IN 47907, USA

**Keywords:** AGASAC, perianal tumor, canine cancer, toceranib, chemotherapy, hypofractionated radiotherapy

## Abstract

**Simple Summary:**

This study evaluates a radiotherapy treatment for anal sac tumors in dogs. The treatment consists of five doses of 4 Gy given consecutively, either alone or combined with other therapies. Our primary objective is to compare the survival and prognostic metrics among patients. Records from fifty dogs were analyzed, showing that those receiving radiotherapy alone (n = 22) had a median survival of 384 days, and a progression free interval of 337 days vs. 628 and 402 days, respectively, from radiotherapy combined with other therapies (n = 28). The treatment caused mild side effects and rare late effects. Overall, adequate palliation for AGASACA can be expected with radiotherapy alone via this dose with acceptable toxicities, and extended survival is possible when combined with other treatments with acceptable toxicities as well.

**Abstract:**

This research aims to evaluate the outcomes of a radiotherapy protocol, consisting of five fractions of 4 Gy each, resulting in a total dose of 20 Gy for apocrine gland anal sac tumors and local lymph nodes in canines. This protocol was assessed as a palliative treatment for macroscopic tumors alone, or in combination with additional therapies under different scenarios. Medical records from fifty canine patients met the inclusion criteria and were divided into different treatment groups: radiotherapy alone (n = 22, 44%), radiotherapy with chemotherapy or targeted therapy with toceranib (n = 18, 36%), surgery with radiotherapy (n = 5, 10%), and surgery with radiotherapy and chemotherapy or targeted therapy with toceranib (n = 5, 10%). Patients who received radiotherapy alone had a median survival time of 384 days (95% CI 198–569) and 628 days (95% CI 579–676) for RT + additional therapies. The median time to progression for patients with radiotherapy alone was 337 days (95% CI 282–391 days), and 402 days (95% CI 286–517 days) for radiotherapy plus additional treatments. Acute side effects were mild, with the majority having diarrhea (61%), and only one patient developed grade III late effects VRTOG v2 classification; however, this happened 22 months after the first radiotherapy protocol after re-irradiation. The results demonstrate that radiotherapy alone under this protocol provided a comparable median time to progression vs. radiotherapy plus additional treatments while maintaining acceptable side effects. The combination of this protocol with other treatment modalities offers attractive results for local disease control and survival while maintaining acceptable toxicities. Overall, these findings contribute to the growing evidence supporting the role of radiotherapy in managing apocrine gland anal sac adenocarcinoma in dogs.

## 1. Introduction

Studies on apocrine gland anal sac adenocarcinoma (AGASACA) in dogs date back more than 50 years [1,2]. Early clinical characterization of the disease has described features of aggressiveness by spreading to the lymph nodes and later to the lungs, while also demonstrating relatively prolonged survival with treatment [3]. Articles have reported outcomes of treatments involving surgery to the primary tumor and/or regional metastasis, with or without adjuvant cytotoxic agents [4,5,6]. However, surgery or chemotherapy may not always be best suited for all patients and owners [6,7,8].

The use of radiotherapy for pelvic irradiation raised concerns about possible life threatening late effects, such as intestinal perforation, incontinence, and skin ulceration, among others [9]. However, studies have highlighted its feasibility with limited risk for the patient [6,10]. Precautions, such as implementing smaller fraction sizes to minimize potential side effects, have been employed [11]. Although clinicians have also evaluated the feasibility of using hypofractionationated protocols with fractions above 4 Gray (Gy) [12,13,14]. These studies have yielded interesting results in terms of survival, with acceptable levels of early and late side effects [12,13,14]. While ongoing interest in definitive protocols remains, the benefits of hypofractionation for palliative intent are undeniably attractive, requiring fewer anesthesia sessions for the patient and less hospitalization [15]. Overall, progress in radiation technology has positioned the hypofractionation modality as a potential option for managing AGASACA [16].

There is currently no gold standard approach for treating AGASACA tumors in dogs [17]. Hence, various radiation therapy fractionation protocols have been reported in the literature [6,9,10,11,12,13,14,15,17]. Of particular interest is a protocol consisting of five fractions of 4 Gy each, administered consecutively to achieve a total dose of 20 Gy. This approach has demonstrated interesting results in various tumor types, offering potential radiobiological benefits while providing adequate palliation [18]. Despite this, only small cohorts of AGASACA patients have been reported with this treatment regimen [18]. Thus, this study aims to present outcomes from a large cohort of patients treated with the above radiotherapy protocol, either as a standalone treatment or in combination with other therapy modalities. Our primary objective was to compare the survival and prognostic metrics among patients. We hypothesized that AGASACA patients treated with this radiotherapy dose can experience survival and prognosis comparable to other protocols and treatment modalities, such as surgery and chemotherapy, while maintaining acceptable side effects for adequate palliation [6,7,8,9,10,11,12,13,14,15,17].

## 2. Materials and Methods

### 2.1. Study Design and Case Selection

This study was designed as a retrospective cohort study; we retrieved patients’ medical data from electronic medical records at Purdue University, College of Veterinary Medicine, Veterinary Teaching Hospital from January 2009 to July 2022. Dogs were eligible for inclusion in the study if they had a confirmed diagnosis of apocrine gland anal sac adenocarcinoma by cytology or pathology by a board-certified pathologist. Additionally, all dogs received a radiotherapy treatment consisting of five daily consecutive fractions of 4 Gy, resulting in a total dose of 20 Gy. The radiation plan and delivery were overseen on-site by a board-certified veterinary radiation oncologist. Dogs that did not meet these criteria were excluded from the study.

Our primary objective was to compare the survival and prognostic metrics among patients receiving the above radiotherapy protocol, alone or in combination with other treatments. We hypothesized that AGASACA patients treated with this radiotherapy dose can experience survival and prognosis comparable to other protocols and treatment modalities, while maintaining acceptable side effects for adequate palliation [6,9,10,11,12,13,14,17].

### 2.2. Clinical Study Design

We extracted from electronic medical records patients’ relevant information (age, breed, and sex), including clinical presentation, diagnosis method, tumor characteristics (e.g., location and size of the tumor), presence of circulating high total calcium at initial diagnosis before any type of intervention (total calcium was elected as ionized calcium is not systematically requested at our institution, and it may better characterize the most common initial clinical presentation of the disease at most hospitals or clinics). Relevant radiotherapy information consisted of the treatment start and end dates, the inclusion or exclusion of lymph nodes, any instances of re-treatment if pursued, and the type of treatment. Treatments were performed using 6 MV photon beams (Varian 6EX linear accelerator with 120-leaf multileaf collimator, Varian Medical Systems, Inc., Palo Alto, CA, USA) at a rate of 400 MU/min to deliver 4 Gy in five consecutive fractions for a total dose of 20 Gy. Treatment planning software (Varian Eclipse v. 11.0 treatment, Varian Medical Systems, Palo Alto, CA, USA) with MLC conformation, and MV portal imaging (KODAK ACR—2000i, Onconcepts, Rochester, NY, USA) were inconsistently used. Mainly manual plans were employed, in a two-open field in anterior–posterior/posterior–anterior arrangement, treating at midline from the tumor location with variable field sizes per patient. When applicable, MLC conformation was used to shape to tumor and lymph nodes; in such cases, the tumor was set as the gross tumor volume (GTV), and an expansion from 1–3 mm was used to set a planning target volume (PTV), tight margins were selected with an intent to palliate and not obtain a disease control. When applicable, delineated organs at risk (OAR) were the rectum, intestines, spine cord, and bladder. Both GTV and lymph nodes were treated in the same field when applicable, and no bolus material was used. Additionally, we collected information pertaining to surgery, chemotherapy, and targeted therapies from medical records, consisting of the start and end dates for each treatment and drugs used in the case of systemic therapies. Patients were categorized into five subgroups according to their treatments to meet the study primary objective goals: (1) Radiotherapy (RT) Alone: receiving radiotherapy as the sole treatment; (2) RT + systemic therapy (Syst): receiving radiotherapy with systemic therapy (chemotherapy with or without targeted therapy); (3) RT + targeted therapy (T): receiving radiotherapy with targeted therapy (toceranib) as a standalone therapy; (4) RT + surgery (Sx): receiving both surgery and radiotherapy; and (5) RT + surgery and systemic therapy (All): receiving full combined therapy involving radiotherapy, surgery, and systemic therapy (with or without targeted therapy).

### 2.3. Survival and Prognostic Metrics

The main study outcomes included survival and progression. Median survival time (MST) was defined as the time from the start of radiotherapy until death, and progression-free interval (PFI) was determined as the time from the start of radiotherapy until disease progression, including those who had any intervention before radiotherapy for both metrics. Due to the nature of the study, progression was defined as the clinical growth (or regrowth in surgical cases) of the primary tumor indicated on medical records, clinical growth of regional metastasis, or new regional or distant metastasis indicated on medical records. A patient’s death, whether from natural causes or euthanasia, attributable to the tumor as documented in medical records or confirmed by the referring veterinarian, was regarded as an event. Patients who died from unrelated causes remained alive at the end of the study or were lost to follow-up were censored.

### 2.4. Side Effects

Acute and late effects were determined according to the guidelines by the Veterinary Radiation Therapy Oncology Group v2 (VRTOG v2) [19]. In cases where the medical records did not provide precise VRTOG classifications, the clinical descriptions found within the records were used to estimate the side effects. To ensure the accuracy of the information, we collected follow-up data from the patients through their referring veterinarians using a questionnaire that was filled by them via email; a period of six months was allowed for completion of it. Acute side effects were defined as those occurring within three months after the treatments, while late effects as those manifesting beyond three months following radiotherapy.

### 2.5. Statistical Analysis

Descriptive statistics were reported using counts and percentages for categorical variables and medians and total rages for continuous variables. We estimated the survival and prognostic metrics using the Kaplan–Meier method. Median survival time, median PFI, and corresponding 95% confidence intervals (CIs) were reported. Comparisons of the survival and prognostic curves between treatment groups were performed using log-rank tests. Cox regression was performed for pairwise comparisons following a significant log-rank test.

In addition, we performed baseline comparisons of age, sex, weight, presence of clinical signs at diagnosis, tumor volume, hypercalcemia, tumor location, presence of local metastasis, and presence of distant metastasis between treatment groups. Specifically, we compared each of them between the RT alone group and the RT + additional therapies group using t-tests (or Mann–Whitney tests, if outliers were observed or not normally distributed according to Shapiro–Wilk tests) for continuous covariates and chi-squared tests for categorical covariates. Covariates found to be significant in the univariate analysis were adjusted for Cox regression, and the adjusted hazard ratio and 95% CI were reported. Statistical significance was defined as *p* < 0.05. All data analyses were performed using IBM SPSS Statistics for Windows (Version 29.0. Armonk, NY, USA: IBM Corp.) and descriptive statistics using Excel software 2021 (Microsoft Corporation, Redmond, WA, USA.

## 3. Result 

### 3.1. Patients & Clinical Information

Fifty canine patients met the inclusion criteria for this study. A total of 82% (n = 41) of diagnostic confirmation was determined through pathology evaluation, while only 18% (n = 9) was determined via cytology analysis. In all cases, the diagnosis was consistent with apocrine gland anal sac adenocarcinoma. Among study patients, 62% (n = 31) were castrated males, while 38% (n = 19) were spayed females. The median (range) weight at presentation was 23 kg (5–49 kg), and the median (range) age was 11 years (4–14 years). The majority of cases were mixed-breed dogs 44% (n = 22), followed by the Labrador Retriever (n = 4) and American Cocker Spaniel (n = 3). American Pit Bull Terrier, Border Collie, German Shepherd, Havanese Terrier, and Dachshund each represented two patients. Lastly, American Eskimo, Australian Shepherd, Beagle, Bichon Frise, Cavalier King Charles Spaniel, Goldendoodle, Irish Setter, Poodle (Standard), Rhodesian Ridgeback, Schnauzer, and Shih Tzu were each represented by one patient per breed (Table 1).

At the time of first presentation, 62% (n = 31) of patients reported clinical signs. The most commonly observed clinical sign was perianal swelling (n = 16, 51%). Difficulty with defecation was reported by 38% (n = 12) of the patients. Additionally, 29% (n = 9) of the patients exhibited stool alterations. Polyuria and polydipsia were observed in 19% (n = 6) of the patients. Scooting and lethargy were each reported in 12% (n = 4) of the patients, and anorexia was present in 9% (n = 3) of the patients. For the location of the tumor, 54% (n = 27) were on the right side, 44% (n = 22) were on the left side, and 2% (n = 1) were bilateral. The initial tumor volume was reported in 64% (n = 32) of the patients, with a median (range) tumor volume of 50.41 cm^3^ (0.9–333 cm^3^). Hypercalcemia data was available for 54% (n = 27) of the patients. Unfortunately, in the rest, information was not available at the time of the study data collection and information regarding clinical management was not available either in many cases. However, from medical records’ updates, the majority of them received prednisone or bisphosphonate as treatment. From the available information from these 27 patients, 71% (n = 20) exhibited normal calcium values at the time of initial staging, while only 25% (n = 7) presented with elevated total calcium. The median calcium concentration was 15 mg/dL (11.9–16 mg/dL).

Thirty-eight patients (76%) showed positive findings for regional metastasis: 57% (n = 22) were suspected with computed tomography (CT), 36% (n = 14) via ultrasound, and 5% (n = 2) with abdominal and pelvic radiographs. Cytology exam and confirmation were obtained for only 13% (n = 5), and pathologic confirmation was achieved in only 2% (n = 1). The most commonly affected lymph nodes included the lumbar lymph nodes (medial and internal iliac) and the medial and lateral sacral lymph nodes. At the time of initial diagnosis, only 6% (n = 3) of the patients had distant metastasis. All three patients had pulmonary metastasis, with two cases confirmed through CT and one case confirmed through radiographs. Among these patients, one had additional metastatic lesions in the spleen and liver (assessed via CT), while another had suspected metastasis in the ribs and femur (assessed via radiographs). The median follow-up time (range) after radiotherapy was 330 days (6–4320 days).

### 3.2. Treatments Results

All treatment plans included the regional lymph node bed (lumbar and sacral lymph nodes); lymph nodes received the same dose as the primary target. Most treatment plans were manual plans (74%, n = 37), while 26% (n = 13) were computer plans using Eclipse v11 (3-D plans).

Among the study patients, 44% (n = 22) were in the RT Alone subgroup, 18% (n = 9) in the RT + Syst subgroup, 18% (n = 9) in the RT + T subgroup, 10% (n = 5) in the RT + Sx subgroup, and 10% (n = 5) in the RT + All subgroup. Patients in the RT +T subgroup received toceranib as a standalone therapy after radiotherapy with a median interval (range) of 159 days (93–612 days). The most commonly used systemic therapeutic agent was carboplatin, while other less common drugs included doxorubicin, mitoxantrone, cyclophosphamide, melphalan, and chlorambucil. Four of the patients in the RT + Sx subgroup underwent surgery before radiotherapy, with a median time (range) of 125 days (45–504 days) between surgery and radiotherapy. Only one patient had surgery after radiotherapy (222 days after the initial treatment; this patient was not re-irradiated).

Among the patients in the RT + ALL subgroup, four underwent surgery at first, followed by systemic therapy, and radiotherapy. The median time (range) from surgery to systemic therapy was 20 days (14–433 days), while the median interval (range) between systemic therapy and radiotherapy was 433 days (94–541 days). Only one patient received radiotherapy after surgery (331 days later), and then systemic therapy (324 days later; this patient was not re-irradiated).

In total, nine patients received re-irradiation treatments, following the same protocol as the initial treatment (5 fractions, 4 Gy per fraction, for a total dose of 20 Gy). The median time (range) from the first radiotherapy session to re-irradiation was 329 days (175–794 days). Among these nine patients, one belonged to the RT + All subgroup. Two patients belonged to the RT + Sx subgroup. Four patients belonged to the RT + Syst subgroup, and among them, three had undergone radiotherapy and a combination of different cytotoxic agents, while one received radiotherapy with toceranib. Finally, two patients were in the RT Alone subgroup.

**Figure 1:**
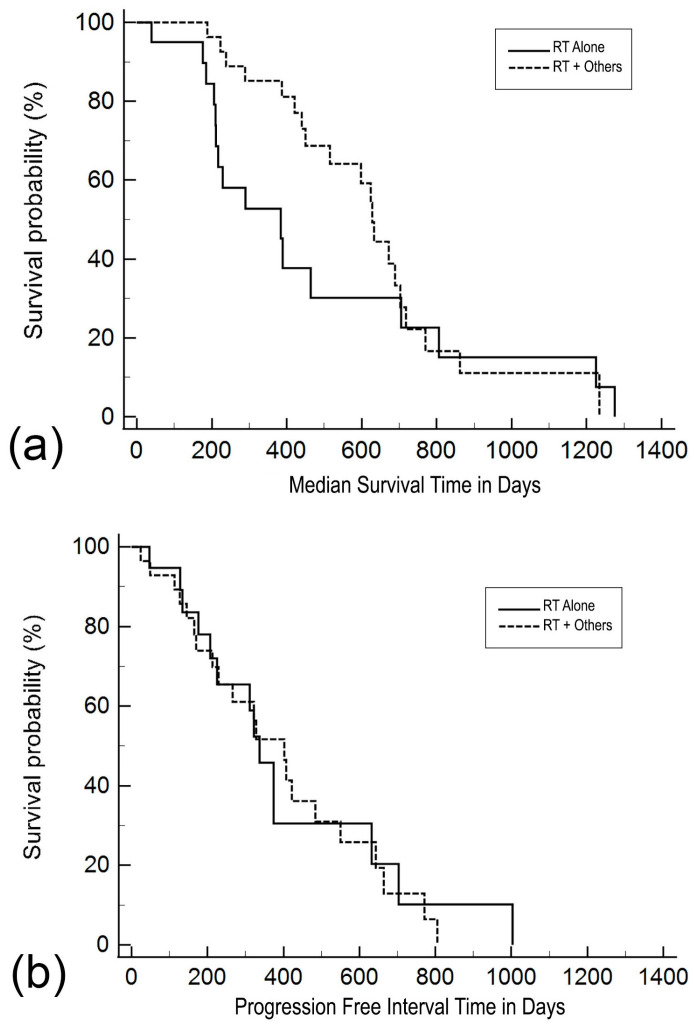
Kaplan–Meier plots for (**a**) median survival time and (**b**) progression-free interval in canine apocrine gland anal sac adenocarcinoma patients. Survival time and progression-free interval are defined as the time from the start of radiotherapy until death and disease progression, respectively. The solid line represents patients treated with radiotherapy as a monotherapy (RT Alone), and the dashed line represents patients treated in a multimodal fashion in which radiotherapy was combined with either surgery, chemotherapy, or targeted therapies (RT + Others).

The median survival time was 384 days (95% CI 198–570) for patients in the RT Alone subgroup and 628 days (95% CI 579–677) for RT + additional therapies (Figure 1a). The median PFI for patients in the RT Alone subgroup was 337 days (95% CI 283–391 days), and for RT + additional therapies, the median was 402 days (95% CI 286–518 days) (Figure 1b).

Comparing the time in the survival curves between different treatment subgroups, the log-rank test did not reveal statistical significance (*p* = 0.814) (Table 2). The protocol involving surgery, systemic therapy, and radiotherapy resulted in the longest median survival time. It is noteworthy that radiotherapy, in combination with a single agent, toceranib (i.e., RT + T subgroup), achieved a comparable median survival time to that of radiation combined with multiple systemic drugs (i.e., RT + Syst subgroup). The survival curves of PFI were also not significantly different between treatment subgroups (*p* = 0.643). However, the RT + T subgroup demonstrated the longest median progression-free interval, reaching 422 days, compared with other subgroups (Table 2).

The baseline comparisons identified the presence of clinical signs at diagnosis as the only covariate significantly different (*p* = 0.049) between RT Alone (77%) and RT + additional therapies (50%). However, adjusting for it (in Cox regression) did not alter the hazard ratio and 95% CI estimations, indicating that the presence of clinical signs at diagnosis was not an influential confounder. Thus, the unadjusted hazard ratios and 95% CIs were reported. The hazard ratios of dying and progression were 1.5 (95% CI: 0.75–3.0, *p* = 0.249) and 0.97 (95% CI: 0.48–1.9, *p* = 0.924), comparing RT Alone to RT + additional therapies.

### 3.3. Side Effects

**Figure 2:**
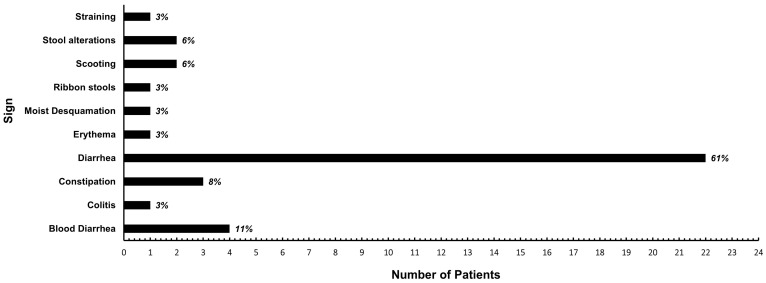
Acute side effects of a palliative treatment of 20 Gy in 5 consecutive fractions protocol for canine apocrine gland anal sac adenocarcinoma patients (n = 50). Acute side effects were considered if the clinical signs appeared no more than 3 months after finishing the radiotherapy protocol. Clinical signs are in alphabetical order. On the right of each bar, a percentage label for that sign is included from the patients that experienced acute side effects (n = 36).

A total of 72% (n = 36) of patients experienced acute side effects. From them, 39% (n = 14, two patients developed gastrointestinal and skin reactions at the same time) belonged to the RT alone group, 22% (n = 8) belonged to the RT + Sys group, 16% (n = 6) belonged to the RT + T group, 11% (n = 4) belonged to the RT + Sx group, and 11% (n = 4) belonged to the RT + All group (Table 3). Among the observed signs, 11% were estimated as grade I (n = 4), 78% as grade II (n = 30), and 11% as grade III (n = 4) according to the VRTOG v2 classification (Figure 2). Diarrhea was the most common side effect reported. All acute side effects resolved with symptomatic therapy and no unexpected complications were encountered, as indicated by the medical records. Information on late effects was available for 68% of the patients (n = 34). Among them, 79% (n = 27) did not experience any late effects following radiotherapy. However, seven patients (21%) did develop late effects. Intermittent diarrhea (grade II VRTOG v2) was the most commonly observed late effect, reported by six of these patients. Additionally, one of the six patients who experienced intermittent diarrhea also developed alopecia (grade I VRTOG v2) in the affected region, belonging to the RT +All group (Table 3). Only one patient (3%) experienced a grade III VRTOG v2 complication, which manifested as non-healing skin ulceration. Of note, the patients who developed skin ulceration received re-irradiation and belonged to the first subgroup of patients who were treated with radiotherapy alone (Table 3). Among the patients who developed chronic diarrhea, half of them underwent re-irradiation. Specifically, one patient received radiotherapy and chemotherapy before re-irradiation, another patient received radiotherapy and toceranib, and one patient received radiotherapy and surgery. The remaining three patients with chronic diarrhea were not re-irradiated. Among them, one patient was treated with radiotherapy and chemotherapy, one with radiotherapy and toceranib, and one with radiotherapy, surgery, and chemotherapy.

## 4. Discussion

When analyzing and comparing with the published outcomes from radiotherapy for AGASACA [6,9,10,11,12,13,14,17], we notice that under certain circumstances, such as when treating primary macroscopic tumors or reactive lymph nodes, the results from conventional fractionation protocols, both in terms of survival and toxicities, can sometimes be similar to what we see for hypofractionation [10,15]. These suggest that hypofractionation could be a treatment option for this type of tumor [20,21]. Our results and clinical experience further support that we can achieve reasonable tumor control under a hypofractionated protocol [15,21]. However, it warrants further confirmation, as there is currently no clear standard for the actual tumor sensitivity to radiation, and most of the series have a limited number of patients; thus, the ideal radiotherapy protocol remains elusive [22]. Nevertheless, it is reasonable to assume that the dose used in this study will not be enough to provide a disease cure in a macroscopic tumor; however, adequate palliation might be achieved [20,21,22].

One important aspect to consider from our retrospective study is the possibility of improving the treatment delivery by adopting newer technologies and treatment planning techniques [20]. Proper radiation conformity is needed to achieve accurate tumor coverage and to reduce side effects for hypofractionated protocols [20,21]. Considering the acute effects observed in our study, lack of conformity due to lack of plan technology likely played a role in their occurrence, as in this case all lymph nodes were treated (lumbar and sacral), a volume of intestines, colon, or the overall pelvic region could have received a higher dose. Therefore, transitioning to technologies such as intensity-modulated radiotherapy with inverse planning, prioritizing high-dose gradients, and inclusion of newer image guidance systems and organs motion consideration, could warrant a decrease in acute side effects [16,23].

Surgery is considered the primary treatment for AGASACA [17,24]; however, it is apparent that specific considerations, such as tumor size, the presence of local metastasis, and lack of iliac vessel invasion, must be taken into account to ensure its success [17,24]. Our results indicate that combining multiple treatment options provides the longest median survival times; however, when analyzing progression-free interval, radiotherapy alone becomes comparable to combining radiotherapy with additional treatments (337 vs. 402 days). In addition, radiotherapy alone might offer comparable median survival times to those achieved when chemotherapy is used alone or when tumor size exceeds ten square centimeters [6]. Our results also provide comparable outcomes from radiotherapy alone when local metastasis is present and treated with surgery, although some authors recommend metastatic lymph node extirpation when possible [8,24,25]. Surgery (with or without systemic therapy, and with or without radiotherapy) by itself can be highly demanding from the perspective of patient care when compared to radiotherapy and may yield comparable benefits as seen in our results, especially for patients with guarded prognoses making hypofractionation radiotherapy an attractive palliation choice [6,7,8,9,10,11,12,13,14,15,17]. Despite the presence of regional metastasis, our findings did not indicate a statistically significant influence on survival metrics. However, this outcome may be subject to bias since all patients in our study received radiotherapy to the lymph nodes, which differs from other studies reporting local metastasis as a negative prognostic marker when not treated [6,7,8], and diagnosis certainty is absent as the majority of lymph nodes metastasis were only confirmed via imaging. Overall, our findings could highlight the potential value of radiotherapy alone as a palliative option for specific clinical presentations (such as when tumor size exceeds ten square centimeters or local metastasis is present) of AGASACA patients.

Existing evidence indicates that AGASACA expresses some of the targets of the tyrosine kinase inhibitor toceranib, including VEGFR, PDGFR, and KIT [26], and has shown favorable responses and prognostic outcomes [26,27,28]. Therefore, an expected potential synergistic effect when combining radiotherapy and toceranib in a proportion of patients could be expected and was indeed observed in our results, challenging the consideration that radiotherapy alone could provide better outcomes to the combination of both. In addition, the radiation and toceranib subgroup presented an overall proportion of late side effects, comparable to the reported morbidity of surgery, ranging from 15% to 20% [8,24]. Hence, combining both treatments could yield adequate palliation with an extended survival while retaining relatively low toxicity in macroscopic tumors, making this combination an attractive option that requires further study.

Re-irradiation case analysis poses several challenges due to the lack of standardized follow-up in this study and the small number (n = 9). In our study, the only grade III late side effect observed was in a patient who was re-irradiated with radiation as the sole therapy. Explanations, such as intrinsic radio sensitivity, focal overdose, or geographic miss are reasonable and suggest that the observed late side effect in this patient may not necessarily be linked with retreatment. In addition, we cannot analyze specific dose points due to the absence of treatment plan usage. Among the six patients who developed chronic diarrhea, three underwent re-irradiation, while the remaining three did not. Furthermore, five re-treated patients did not experience any late side effects. Overall, we cannot provide conclusive evidence for the effects of re-treatment with this small cohort; however, our results might suggest the feasibility of re-treatment with this dose [29,30].

Demographic and clinical data characteristics of our study population were consistent with previous studies [6,17,24]. Which sustain the importance of considering AGASACA in the differential diagnosis of dogs presenting with perianal or anal region abnormalities, including inflammation, pain, difficulty of defecating, or elderly dogs during wellness exams, as some presented without clinical signs [6,17,24]. Hypercalcemia was observed in 25% of the patients at the time of diagnosis, which correlates with the literature data [6,17,24]. There was no statistical significance found between hypercalcemia and survival, which is comparable to other studies [6,31,32].

As a retrospective study, data availability and completeness can be limited, in addition confounding is always a concern in an observational study like this. Although we performed baseline comparisons to identify potential confounders for statistical adjustment, information on some important covariates might have been missing and Type II error might have explained the findings only in one significant covariate. Another source of bias for this study was immortal time bias as patients in different RT + other therapies subgroups would have to survive until the completion of all therapies to be included in this study. This would show bias, favoring those subgroups over the RT Alone subgroups if additional therapies were administered after the RT. Caution is warranted when analyzing treatment responses due to the absence of standardized follow-up procedures and control groups. Lastly, when analyzing side effects, no in-depth analysis per group substage, treatment, or loss to follow-up patients was conducted.

## 5. Conclusions

The results of this study show that patients treated with this radiotherapy protocol combined with additional therapies experience the longest survivals and prognostic metrics. However, this radiotherapy protocol alone can offer attractive and comparable results for specific clinical presentations of AGASACA patients (such as when tumor size exceeds ten square centimeters or local metastasis is present). The possibility of combining this radiotherapy protocol, especially with targeted therapies, can be an appealing option, although further investigation is needed. Overall, adequate palliation for AGASACA can be expected with radiotherapy alone via this dose in a macroscopic setting with acceptable toxicities, and extended survivals can be possible when combined with other treatments with acceptable toxicities as well.

## Figures and Tables

**Table 1:** Clinical characteristics of patients at initial diagnosis (before any intervention) classified as per treatment group.

	All Patients (n = 50, 100%)	RT Alone (n = 22)	RT + Sys (n = 9)	RT + T (n = 9)	RT + Sx (n = 5)	RT + All (n = 5)
Age in years (median; range)	11 (4–14)	11 (7–13)	11 (8–14)	9 (5–14)	10 (4–11)	10 (6–12)
weight in Kg (median; range)	23 (5–49)	25.3 (5–49)	22 (13–37)	23.4 (6–38.5)	22.9 (13–49)	15.2 (11–34.4)
Sex						
FS	19 (38%)	6	4	5	2	2
MN	31 (62%)	16	5	4	3	3
Breed						
American Cocker Spaniel	3 (6%)	1	1	1		
American Eskimo	1 (2%)				1	
American Pit Bull Terrier	2 (4%)	1	1			
Australian Shepherd	1 (2%)		1			
Beagle	1 (2%)					1
Bichon Frise	1 (2%)					1
Border Collie	2 (4%)		1	1		
Cavalier King Charles Spaniel	1 (2%)	1				
Dachshund	2 (4%)			1		1
German Shepherd	2 (4%)			1		1
Goldendoodle	1 (2%)				1	
Havanese Terrier	2 (4%)	2				
Irish Setter	1 (2%)					1
Labrador Retriever	4 (8%)	3			1	
Mixed Breed Dog	22 (44%)	12	5	4	1	
Poodle (Standard)	1 (2%)			1		
Rhodesian Ridgeback	1 (2%)	1				
Schnauzer	1 (2%)	1				
Shih Tzu	1 (2%)	1				
Clinical signs at presentation						
Yes	31 (62%)					
No	19 (38%)					
Anorexia	3 (9%)	2	1			
Difficulty Defecating	12(38%)	7	2	1	1	1
Lethargy	3 (9%)	5				
Perianal Swelling	16 (51%)	10	2	3	1	
Polyuria/Polydipsia	6 (19%)	3	1	1	1	
Scooting	4 (12%)	2		1	1	
Stool alterations	9 (29%)	4	1	2	1	1
Location						
Right	27 (54%)	10	5	6	3	3
Left	22 (44%)	12	4	3	2	1
Bilateral	1 (2%)					1
Tumor Volume in cm^3^ (median; range)	50.41 (0.9–333)	44.87 (0.94–333)	47.7 (6.37–179.5)	62.23 (24.42–200)	49	7.8 (1.54–14.13)
Calcium Levels						
n/a	23 (46%)					
available	27 (54%)	10	4	4	2	3
	normal (20; 71%)	9	3	4	2	2
	hypercalcemic (7; 25%)	3	2	1	1	
Regional metastasis						
Yes	38 (76%)	16	8	8	4	2
No	12 (24%)	6	1	1	1	3
Distant metastasis						
Yes	3 (6%)	1		2		
No	47 (94%)	21	9	7	5	5

RT Alone: radiotherapy only; RT + Syst: radiotherapy with systemic therapy (chemotherapy with or without targeted therapies); RT + T: radiotherapy with targeted therapy as standalone therapy; RT + Sx: radiotherapy and surgery; RT + All: full combined therapy involving radiotherapy, surgery, and systemic therapy; FS: female spayed; MN: male neuter. n/a: not available.

**Table 2:** Median survival time and median progression-free interval, estimated using the Kaplan–Meier method, in canine apocrine gland anal sac adenocarcinoma patients by treatment subgroups. Survival time and progression-free interval are defined as the time from the start of radiotherapy until death and disease progression, respectively.

	RT Alone (n = 22)	RT + Sys (n = 9)	RT + T (n = 9)	RT + Sx (n = 5)	RT + All (n = 5)
	Survival time (in days)				
Median	384	672	628	450	703
95% CI	198–570	558–786	269–987	315–585	na
	Progression-free interval (in days)				
Median	337	266	422	402	na
95% CI	283–391	196–336	100–744	88–716	na

RT Alone: radiotherapy only; RT + Syst: radiotherapy with systemic therapy (chemotherapy with or without targeted therapies); RT + T: radiotherapy with targeted therapy as standalone therapy; RT + Sx: radiotherapy and surgery; RT + All: full combined therapy involving radiotherapy, surgery, and systemic therapy. na: insufficient number of patients to derive the estimation. CI: confidence interval.

**Table 3:** Number of patients per treatment group that developed acute and late side effects according to the Veterinary Radiation Therapy Oncology Group v2 (VRTOG v2).

			RT Alone (n = 14)	RT + Sys (n = 8)	RT + T (n = 6)	RT + Sx (n = 4)	RT + All (n = 4)
Acute Side Effects (36 patients; 72%)	Skin	Grade I	1 (2%)				
		Grade II	1 (2%)				
	Gastrointestinal	Grade I	2 (5%)	2 (5%)		1 (3%)	1 (3%)
		Grade II	10 (27%)	6 (17%)	4 (11%)	3 (8%)	3 (8%)
		Grade III	2 (5%)		2 (5%)		
			RT Alone (n = 1)	RT + Sys (n = 2)	RT + T (n = 2)	RT + Sx (n = 1)	RT + All (n = 2)
Late Side Effects (7 patients; 21%)	Skin	Grade I					1 (3%)
		Grade II					
		Grade III	1 (3%)				
	Gastrointestinal	Grade I					
		Grade II		2 (6%)	2 (6%)	1 (3%)	1 (3%)
		Grade III					

RT Alone: radiotherapy only; RT + Syst: radiotherapy with systemic therapy (chemotherapy with or without targeted therapies); RT + T: radiotherapy with targeted therapy as standalone therapy; RT + Sx: radiotherapy and surgery; RT + All: full combined therapy involving radiotherapy, surgery, and systemic therapy. For acute side effects, data were available from all patients (n = 50); in the RT Alone group, two patients developed gastrointestinal and skin reactions at the same time, and thus they are included as one patient each (n = 14; 39%). For late effects, information was available from 34 patients (n = 34).

## Data Availability

The data are not publicly available to ensure compliance with privacy and confidentiality policies governing veterinary patient information at Purdue University’s veterinary teaching hospital. Interested parties may contact the corresponding author to discuss potential access to the data, subject to compliance with relevant ethical and legal considerations.
